# Signature of survival: a ^18^F-FDG PET based whole-liver radiomic analysis predicts survival after ^90^Y-TARE for hepatocellular carcinoma

**DOI:** 10.18632/oncotarget.23423

**Published:** 2017-12-19

**Authors:** Paul Blanc-Durand, Axel Van Der Gucht, Mario Jreige, Marie Nicod-Lalonde, Marina Silva-Monteiro, John O. Prior, Alban Denys, Adrien Depeursinge, Niklaus Schaefer

**Affiliations:** ^1^ Department of Nuclear Medicine and Molecular Imaging, Lausanne University Hospital, Lausanne, Switzerland; ^2^ Department of Radiology and Interventional Radiology, Lausanne University Hospital, Lausanne, Switzerland; ^3^ Institute of Information Systems, University of Applied Sciences Western Switzerland [HES-SO], Sierre, Switzerland

**Keywords:** ^18^F-FDG PET, TARE, radiomics, hepatocellular carcinoma, survival

## Abstract

**Purpose:**

To generate a predictive whole-liver radiomics scoring system for progression-free survival (PFS) and overall survival (OS) in patients undergoing transarterial radioembolization using Yttrium-90 (^90^Y-TARE) for unresectable hepatocellular carcinoma (uHCC).

**Results:**

The generated pPET-RadScores were significantly correlated with survival for PFS (median of 11.4 mo [95% confidence interval CI: 6.3–16.5 mo] in low-risk group [PFS-pPET-RadScore < 0.09] vs. 4.0 mo [95% CI: 2.3–5.7 mo] in high-risk group [PFS-pPET-RadScore > 0.09]; *P* = 0.0004) and OS (median of 20.3 mo [95% CI: 5.7–35 mo] in low-risk group [OS-pPET-RadScore < 0.11] vs. 7.7 mo [95% CI: 6.0–9.5 mo] in high-risk group [OS-pPET-RadScore > 0.11]; *P* = 0.007). The multivariate analysis confirmed PFS-pPET-RadScore (*P* = 0.006) and OS-pPET-RadScore (*P* = 0.001) as independent negative predictors.

**Conclusion:**

Pretreatment ^18^F-FDG PET whole-liver radiomics signature appears as an independent negative predictor for PFS and OS in patients undergoing ^90^Y-TARE for uHCC.

**Methods:**

Pretreatment ^18^F-FDG PET of 47 consecutive patients undergoing ^90^Y-TARE for uHCC (31 resin spheres, 16 glass spheres) were retrospectively analyzed. For each patient, based on PET radiomics signature from whole-liver semi-automatic segmentation, PFS and OS predictive PET-radiomics scores (pPET-RadScores) were obtained using LASSO Cox regression. Using X-tile software, the optimal score to predict PFS (PFS-pPET-RadScore) and OS (OS-pPET-RadScore) served as cutoff to separate high and low-risk patients. Survival curves were estimated using the Kaplan-Meier method. The prognostic value of PFS and OS-pPET-RadScore, Barcelona-Clinic Liver Cancer staging system and serum alpha-fetoprotein level was analyzed to predict PFS and OS in multivariate analysis.

## INTRODUCTION

Hepatocellular carcinoma (HCC) is responsible for significant morbidity and mortality. It is the most common primary liver cancer and represents the 2nd most common cause of cancer mortality worldwide [1]. The identification of accurate predictive factors to guide therapy was subject of numerous studies and several robust predictors of death as portal vein invasion (PVI), tumor size, serum alpha-fetoprotein (AFP) level, Child-Pugh class [2], the tumor-node-metastasis (TNM), the Okuda [3] and Barcelona-Clinic Liver Cancer (BCLC) systems [4] or the Cancer of the Liver Italian Program (CLIP) score [5] have been described. In many of these factors, imaging is essential and therefore plays an important role in the management. Multiple studies have shown a correlation between standardized uptake value (SUV) of HCC on ^18^F-fluorodeoxyglucose positron emission tomography (^18^F-FDG PET) and outcomes following different systemic and locoregional treatments [6–16], including more recently transarterial radioembolization with Yttrium-90 (^90^Y-TARE) [17–19].

Recently, radiomics has been introduced in the field of oncology [20]. Radiomics is a fast evolving medical field consisting in the extraction of high-throughput quantitative imaging features that may quantify *in vivo* and noninvasively intra and inter- tissue textural heterogeneity [21]. Indeed, radiomics allows virtual biopsies [20] that captures the inner organization processes of an entire volume with the surrounding tissue without being limited to the sampling site contrary to conventional biopsies. Additionally, virtual biopsies are noninvasive, instantaneous, can be repeated over the time and permit the monitoring of the host tumor relationships and of the treatment sequence. Radiomics does not have a consensual definition but its aim is to provide a characterization of images phenotypes [22] using extracted parameters from medical images (often more than 200+ features [21]) which can be used as biomarkers. They may include first order statistic (intensity, histogram analysis), shape (such as sphericity), textural features (sometimes intensity features or shape are confounded with textural features) or wavelets decompositions. The emerging field of radiomics have sparked large interest the past few years for different imaging modalities (computed tomography [CT], magnetic resonance imaging [MRI], PET) and many cancers such as esophagus, non-small cell lung cancer [21–23] or breast cancer [24].

For the HCC, interest of radiomics has already been reported. Using an integrated imaging-genomic approach with semiquantitative CT features relative to the poorly defined tumor margin, Kuo *et al*. were able to identify HCC imaging phenotypes at CT that correlate with a doxorubicin drug response gene expression program [25]. In another study Segal *et al*. [26] demonstrated that combinations of 28 imaging phenotypes can reconstruct 78% of the global gene expression programs of primary human liver cancer.

Despite the growing evidence for radiomics, no predictive studies in HCC using this technique exist. The aim of the current study was to generate a predictive PET radiomics scoring system for progression-free survival (PFS-pPET-RadScore) and overall survival (OS-pPET-RadScore) in patients undergoing ^90^Y-TARE for unresectable HCC (uHCC) using a pretreatment ^18^F-FDG PET whole-liver radiomics signature. When compared to the previous studies listed above, we used intensity and texture analyses of the entire liver volume, providing an advanced signature of the metabolic heterogeneity and morphology for the subtle distinction of HCC and liver cirrhosis.

## RESULTS

### Patients and subgroups characteristics

The characteristics of the whole-population and low-risk and high-risk groups are given in Table 1. Data of ^90^Y-TARE and associated treatments are given in Table 2. The mean interval between ^18^F-FDG PET/CT and ^90^Y-TARE was 18 days (range, 1–85 days). Patients did not receive any treatment between ^18^F-FDG PET/CT and ^90^Y-TARE. Using the BCLC staging system, 3 patients (6.5%) were stage A, 18 (38.5%) stage B and 26 (55%) stage C. Three patients had normal livers, all others (94%) had cirrhotic liver disease including 36 patients Child-Pugh A and 8 patients Child-Pugh B (≤ B7). Two patients have periportal lymphadenopathy. Among the 47 patients, 19 (40%) were treatment naïve and 28 (60%) had already received various procedures before ^90^Y-TARE including targeted therapy by Sorafenib or Everolimus with an association of 2 or more treatment modalities in 7 patients (15%). With regards to the comparison between low-risk and high-risk groups, the analysis revealed a significant higher tumor size in the high-risk group for OS-pPET-RadScore (*P* = 0.02). A trend for higher tumor size was seen for PFS-pPET-RadScore (*P* = 0.05). The hepatic control rate at 6 months of lesions treated by ^90^Y-TARE was better (but not statistically significant probably explained by the limited number of patients) in low-risk group compared to high-risk group for both PFS-pPET-Radscore (76 vs. 60%; *P* = 0.43) and OS-pPET-Radscore (79 vs. 64; *P* = 0.29).

**Table 1:** Characteristics of the whole-population and low-risk and high-risk groups based on PFS and OS-pPET-RadScores

Characteristics	All patients	PFS-pPET-RadScore			OS-pPET-RadScore		
		Low-risk group	High-risk group	*P*	Low-risk group	High-risk group	*P*
Subjects	47	42	5		33	14	
Cutoff pPET-RadScore	NA	<0.09	>0.09		<0.11	>0.11	
Age, years	68 (61–73)	68 (62–72)	73 (51–75)	0.64	69 (66–72)	63 (55–74)	0.31
Female	6 (12.8)	5 (11.9)	1 (20)	0.61	4 (12.1)	2 (14.3)	0.84
Comorbidities							
Hypertension	17 (36.2)	16 (38.1)	1 (20)	0.43	13 (39.4)	4 (28.6)	0.48
Type 2 diabetes mellitus	15 (31.9)	15 (35.7)	0 (0)	0.11	13 (39.4)	2 (14.3)	0.09
Coronary artery disease	6 (12.8)	6 (14.3)	0 (0)	0.37	5 (15.2)	1 (7.1)	0.45
HCC characteristics							
Tumor size, cm	6.0 (4.3–9.0)	5.5 (3.8–8.4)	8.9 (8.6–9.4)	0.05	4.8 (3.4–7)	8.8 (6.2–11)	**0.02**
<5 cm	19 (40.4)	19 (45.2)	0 (0)	0.05	17 (51.5)	2 (14.3)	**0.02**
≥5 cm	28 (59.6)	23 (54.8)	5 (100)	0.05	16 (48.5)	12 (85.7)	**0.02**
Uni-nodular	24 (51.1)	21 (50)	3 (60)	0.67	14 (42.4)	10 (71.4)	0.07
Multi-nodular (2–5 nodules)	5 (10.6)	5 (11.9)	0 (0)	0.41	5 (15.2)	0 (0)	0.12
Diffuse (> nodules)	18 (38.3)	16 (38.1)	2 (40)	0.93	14 (42.4)	4 (28.6)	0.37
PVI	21 (44.7)	20 (47.6)	1 (20)	0.24	14 (42.4)	7 (50)	0.63
Serum AFP level, kUI/l	17 (6–192)	15 (6–124)	1507 (6–4430)	0.42	12.7 (5-81)	119 (6–3699)	0.37
BCLC staging system							
Stage A	3 (6.4)	3 (7.1)	0 (0)	0.54	3 (9.1)	0 (0)	0.24
Stage B	18 (38.3)	17 (40.5)	1 (20)	0.37	14 (42.4)	4 (28.6)	0.37
Stage C	26 (55.3)	22 (52.4)	4 (80)	0.24	16 (48.5)	10 (71.4)	0.15
Ascites	7 (14.9)	7 (16.7)	0 (0)	0.32	5 (15.2)	2 (14.3)	0.94
Cirrhosis	44 (93.6)	40 (95.2)	4 (80)	0.19	32 (97)	12 (85.7)	0.15
Child-Pugh score A	36 (76.6)	33 (78.6)	3 (60)	0.35	27 (81.8)	9 (64.3)	0.19
Child-Pugh score B (≤B7)	8 (17)	7 (16.7)	1 (20)	0.85	5 (15.2)	3 (21.4)	0.60
Chronic alcoholism	24 (51.1)	22 (52.4)	2 (40)	0.60	17 (51.5)	7 (50)	0.92
Viral infection type B, C	17 (36.2)	16 (38.1)	1 (20)	0.43	12 (36.4)	5 (35.7)	0.97
Hemochromatosis	2 (4.3)	2 (4.8)	0 (0)	0.62	2 (6.1)	0 (0)	0.35
NASH	5 (10.6)	5 (11.9)	0 (0)	0.41	5 (15.2)	0 (0)	0.12

Values are median (25^th^; 75^th^ interquartile range) or *n* (%). AFP, alpha-fetoprotein; HCC, hepatocellular carcinoma; BCLC, barcelona-clinic liver cancer; PVI, portal vein invasion; NASH, non-alcooholic steatohepatitis; TARE, transarterial radioembolization

**Table 2:** ^90^Y-TARE and treatment associated data

Characteristics	All patients	PFS pPET-RadScore			OS-pPET-RadScore		
		Low-risk group	High-risk group	*P*	Low-risk group	High-risk group	*P*
Subjects, *n*	47	42	5		33	14	
^90^Y-TARE data							
^90^Y-resin	31 (66)	27 (64.3)	4 (80)	0.48	19 (57.6)	12 (85.7)	0.06
^90^Y-glass	16 (34)	15 (35.7)	1 (20)	0.48	14 (42.4)	2 (14.3)	0.06
^90^Y-administered activity, GBq	1.6 (1.2–2.5)	1.5 (1.1–2.1)	2.4 (1.6–2.5)	0.26	1.5 (1.1–2.0)	1.9 (1.5–2.6)	0.22
TV based on 99mTc-MAA SPECT/CT, cm^3^	170 (80–615)	157 (80–600)	430 (220–560)	0.65	150 (60–300)	435 (148–646)	0.15
^90^Y-administered activity per unit of TV, MBq/cm^3^	7.3 (4.9–16.2)	7.9 (4.9–16.3)	6.4 (5.6–7.3)	0.81	9.8 (5.2–16.7)	6.0 (3.9–8.4)	0.31
^90^Y-tumor liver absorbed dose, Gy	170 (115–281)	170 (114–286)	205 (155–230)	0.86	170 (115–290)	159 (119–224)	0.91
^90^Y-normal liver absorbed dose, Gy	40 (22–63)	40 (21–64)	36 (30–54)	0.33	43 (24–70)	33 (23–50)	0.05
Unilobar	26 (55.4)	24 (57.1)	2 (40)	0.47	20 (60.6)	6 (42.9)	0.26
Bilobar	8 (17)	6 (14.3)	2 (40)	0.15	5 (15.2)	3 (21.4)	0.60
Segmental	8 (17)	7 (16.7)	1 (20)	0.85	5 (15.2)	3 (21.4)	0.60
Lobar and segmental	5 (10.6)	5 (11.9)	0 (0)	0.41	3 (9.1)	2 (14.3)	0.60
Treatments pre-^90^Y-TARE							
Targeted therapy (Sorafenib)	4 (8.5)	3 (7.1)	1 (20)	0.45	3 (9.1)	1 (7.1)	0.55
Embolization	3 (6.4)	3 (7.1)	0 (0)	0.54	3 (9.1)	0 (0)	0.24
TACE	11 (23.4)	10 (23.8)	1 (20)	0.85	9 (27.3)	2 (14.3)	0.34
Radiofrequency ablation	6 (12.8)	6 (14.3)	0 (0)	0.37	5 (15.2)	1 (7.1)	0.45
Ethanol ablation	3 (6.4)	3 (7.1)	0 (0)	0.54	2 (6.1)	1 (7.1)	0.24
^90^Y-TARE	1 (2.1)	1 (2.4)	0 (0)	0.73	1 (3)	0 (0)	0.51
Treatments after ^90^Y-TARE							
Targeted therapy (Sorafenib)	4 (8.5)	3 (7.1)	1 (20)	0.33	3 (9.1)	1 (7.1)	0.83
Embolization	1 (2.1)	1 (2.4)	0 (0)	0.73	1 (3)	0 (0)	0.51
TACE	10 (21.3)	10 (23.8)	0 (0)	0.22	9 (27.3)	1 (7.1)	0.12
Radiofrequency ablation	7 (14.9)	7 (16.7)	0 (0)	0.32	6 (18.2)	1 (7.1)	0.33
Ethanol ablation	1 (2.1)	1 (2.4)	0 (0)	0.73	1 (3)	0 (0)	0.51
Hepatectomy	1 (2.1)	1 (2.4)	0 (0)	0.73	1 (3)	0 (0)	0.51
^90^Y-TARE	1 (2.1)	1 (2.4)	0 (0)	0.73	1 (3)	0 (0)	0.51

Values are median (25^th^; 75^th^ interquartile range) or *n* (%).

TV, tumor volume; TARE, transarterial radioembolization; TACE, transarterial chemoembolization; SPECT/CT, single-photon emission computed tomography 99mTC-MAA, technetium-macroaggregated albumin.

### Construction of PFS and OS-pPET-RadScores

**Figure 1:**
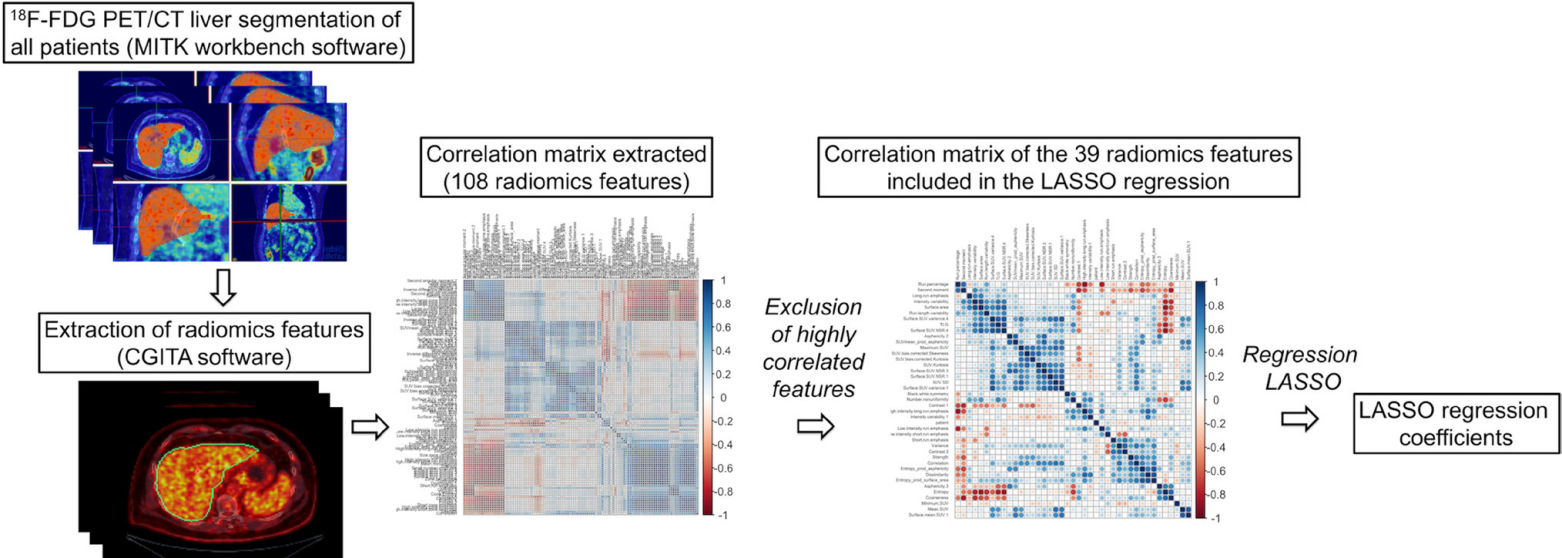
Steps of radiomics process

**Figure 2:**
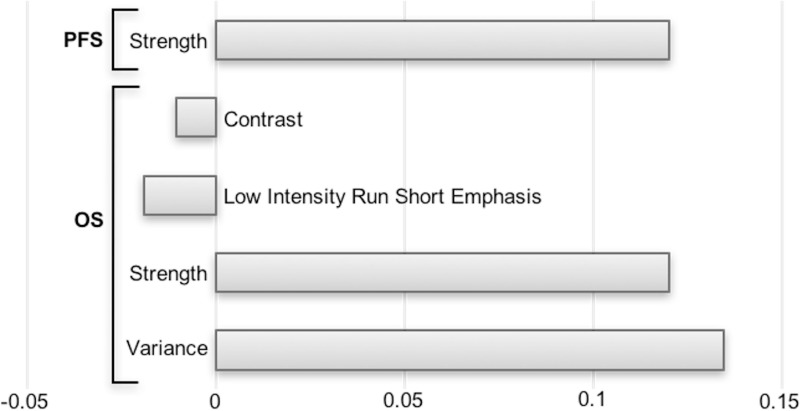
Histogram of the LASSO regression coefficients used to generate PFS and OS-pPET-RadScores

As shown in Figure 1, out of a total 108 radiomics features, 69 were highly correlated and were excluded from the analysis. On the 39 remaining features, a LASSO Cox regression analysis was performed to assess variables with non-zero coefficients. The contribution of the selected parameters with their regression coefficient for the radiomics signature construction is illustrated in Figure 2 by a histogram which shows the importance of each regression coefficients used to generate PFS and OS-pPET-RadScores. PFS and OS-pPET-RadScores were calculated using regression coefficients from the LASSO regression as follow:

$$\begin{array}{ll} \text{PFS PETRadScore} & =0.1201883*\text{Strength}\\ \text{OS PETRadScore} & =0.13444452*\text{Variance}+0.12018832*\text{Strength}\\ & -0.01887273*\text{Low Intensity Run Short Emphasis}\\ & -0.01046038*\text{Contrast} \end{array}$$

### Survival analysis

**Figure 3:**
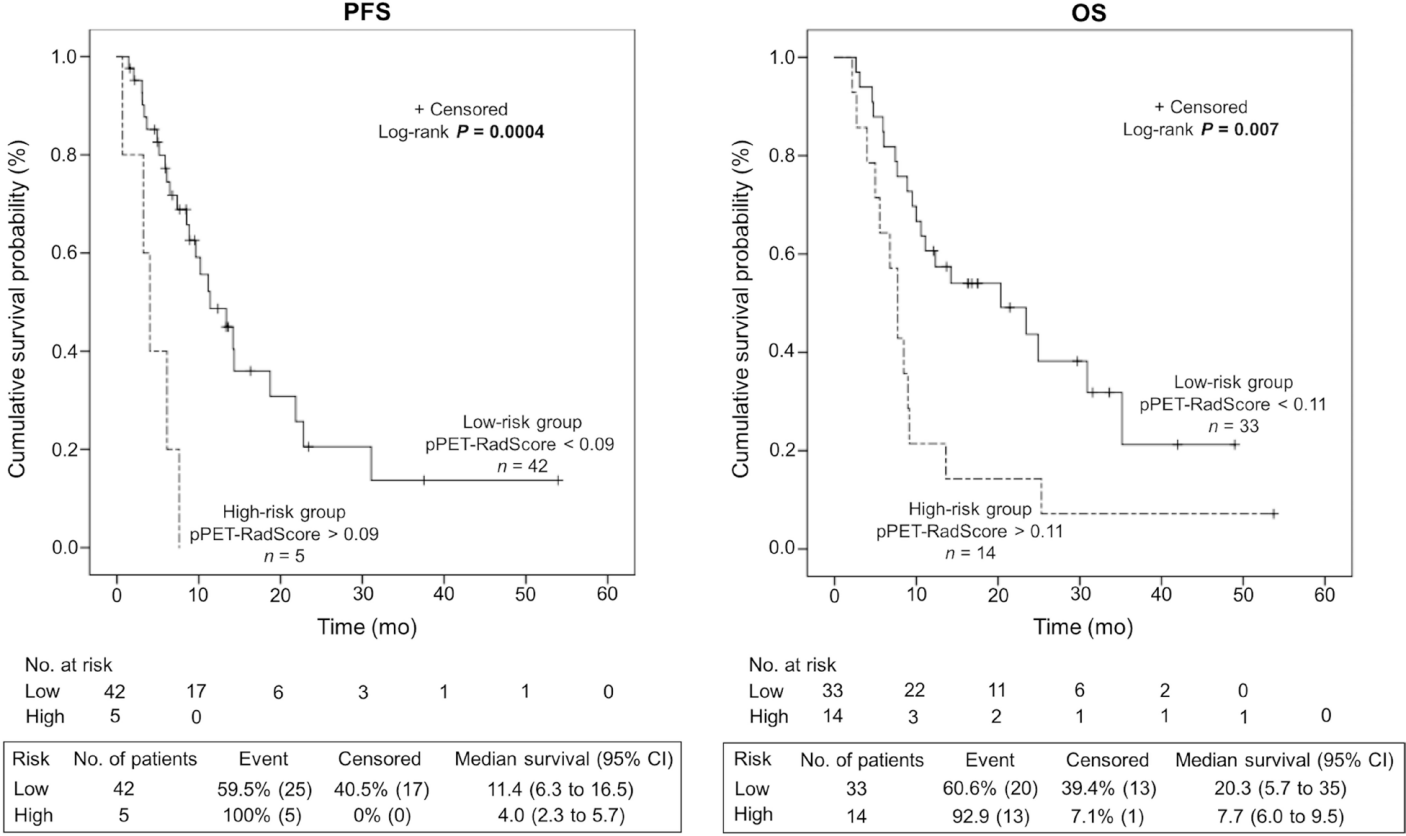
Kaplan-Meier estimates of progression-free survival (PFS) and overall survival (OS) according to the risk profile based on PFS and OS-pPET-RadScores

The median duration of follow-up was 11.1 mo (range, 2.2–53.7 mo). Hepatic relapse occurred in 30 patients (64%) at a median of 6.9 mo (range, 0.7–31.1 mo) after ^90^Y-TARE and 33 (70%) patients died from tumor progression. As shown in Figure 3, the generated pPET-RadScores were significantly correlated with survival for PFS (median of 11.4 mo [95% confidence interval CI: 6.3–16.5 mo] in low-risk group [PFS-pPET-RadScore < 0.09] vs. 4.0 mo [95% CI: 2.3–5.7 mo] in high-risk group [PFS-pPET-RadScore > 0.09]; *P* = 0.0004) and OS (median of 20.3 mo [95% CI: 5.7–35 mo] in low-risk group [OS-pPET-RadScore < 0.11] vs. 7.7 mo [95% CI: 6.0–9.5 mo] in high-risk group [OS-pPET-RadScore > 0.11]; *P* = 0.007). The multivariate analysis confirmed PFS-pPET-RadScore (hazard ratio [HR]: 120, 95% CI: 3.98–3625; *P* = 0.006) and OS-pPET-RadScore (HR: 16.1, 95% CI: 2.94–88.3; *P* = 0.001) as independent negative predictors. The prognostic value of generated PFS and OS-pPET-RadScores did not differ when stratified by BCLC staging system or tumor size (Table 3).

**Table 3:** Multivariable regression for PFS and OS

PFS			OS		
Characteristics	HR (95% CI)	*P*	Characteristics	HR (95% CI)	*P*
PFS-pPET-RadScore	120 (3.98–3625)	**0.006**	OS-pPET-RadScore	16.1 (2.94–88.3)	**0.001**
BCLC staging system			BCLC staging system		
Stages A vs. B	0.57 (0.07–4.65)	0.60	Stages A vs. B	1.31 (0.28–6.18)	0.73
Stages A vs. C	0.72 (0.33–1.59)	0.42	Stages A vs. C	0.66 (0.31–1.42)	0.29
Serum AFP level	0.98 (0.68–1.40)	0.89	Serum AFP level	0.75 (0.45–1.26)	0.28
Size <5 vs. ≥5 cm	0.87 (0.37–2.02)	0.75	Size <5 vs. ≥5 cm	1.0 (0.45–2.26)	0.99
Stratified for BCLC staging system					
PFS pPET-RadScore	92.2 (2.91–2916)	**0.01**	OS-pPET-RadScore	24.8 (3.78–162)	**0.001**
Serum AFP level	0.99 (0.69–1.40)	0.93	Serum AFP level	0.76 (0.45–1.28)	0.30
Size < 5 vs. ≥ 5 cm	0.86 (0.36–2.07)	0.73	Size < 5 vs. ≥ 5 cm	0.89 (0.39–2.04)	0.79
Stratified for tumor size					
PFS-pPET-RadScore	146 (3.97–5333)	**0.007**	OS-pPET-RadScore	16.97 (2.90–99.3)	**0.002**
Serum AFP level	0.96 (0.67–1.38)	0.83	Serum AFP level	0.75 (0.45–1.26)	0.28
BCLC staging system			BCLC staging system		
Stages A vs. B	0.64 (0.08–5.44)	0.68	Stages A vs. B	1.0 (0.21–4.72)	1.00
Stages A vs. C	0.76 (0.34–1.70)	0.50	Stages A vs. C	0.57 (0.26–1.22)	0.15

AFP, alpha-fetoprotein; HR, hazard ratio; CI; confidence interval; PFS, progression-free survival; OS, overall survival; BCLC, barcelona-clinic liver cancer.

## DISCUSSION

The aim of the current study was to generate a predictive radiomics scoring system based on the whole-liver (tumor and non-tumoral liver) segmentation of ^18^F-FDG PET in patients undergoing ^90^Y-TARE for uHCC. This study which describes whole-liver (tumor and non-tumoral) radiomics, might be an interesting concept to integrate liver function and tumor biology. This integrative model may be able to separate patients in low-risk and high-risk groups and to predict survival. This is of interest since ^90^Y-TARE is costly and sometimes associated with side effects in this vulnerable patient population. By introducing the whole-liver and not isolated tumors in the radiomics model, we aim to integrate liver function and tumor biology, thus representing the liver biology in one system. This approach in our view might represent the fragile balance between HCC and liver cirrhosis.

Two predictive radiomics scores were generated to predict survival. These radiomics scores successfully classified patients between low-risk and high-risk in either PFS and OS and remains statistically significant in the multivariate analysis independently of the BCLC staging system which includes variables related to tumor stage, liver functional status, performance status, and cancer-related symptoms [4]. Our score furthermore remained an independent factor against the tumor size and the AFP level in our patient population.

Comparison between low-risk and high-risk revealed that a higher tumor size was seen in the high-risk group for the PFS-pPET-RadScore and OS-pPET-RadScore. Indeed, the tumor size is a well-known factor associated with outcome. Interestingly, our approach replaces the tumor size and functional parameters of the BCLC classification as performance status, PVI and Child-Pugh score with a whole-liver radiomics approach taking into account the lesion size as well as the metabolic activity of the non-tumoral but cirrhotic liver. The generated radiomics score of this study remained significant in the multivariate analysis, mandating an independent value of our mathematical model. Furthermore, a whole-liver radiomics model is more less prone to failure due to lesion interpretation by the radiologist/nuclear physician and can capture much more complex patterns than reported by the BCLC scoring system. The mandated segmentation can be performed easily on the CT scan and then translated on the ^18^F-FDG PET images. Furthermore, in the future, this process of segmentation will be even more fast, reproducible and user-friendly with fully automated liver segmentation integrated into clinical routine [27]. Finally, the segmentation of the whole-liver metabolism could have an additional potential clinical significance if a predictive model of toxicity were identified in future studies.

The main textural features in our predictive radiomics scoring system were Strength and Variance. The presence of the Strength in both PFS and OS models confirmed the relevant predictive value of this parameter. Strength is a textural feature based on the neighborhood gray-tone difference matrix that is first described in 1989 by Amadasun *et al*. [28] and means if a pattern is perceivable within the texture and if it can be recognized. Variance is a textural feature derived from texture feature coding method and describes a deviation from the mean of textural feature numbers (a transformation of the image voxels that represent a certain type of local texture). Variance is one of the textural feature that was initially used by Horng *et al*. to classify ultrasonic liver images into 3 liver states (normal liver, hepatitis and cirrhosis) with a correct classification rate of 86.7% and a false-negative rate of 4.4% [29]. We believe that this publication strengthens our integrative whole-liver approach using radiomics and emphasizes once more the importance to include not only tumor lesions to predict outcome but also have to a tool to assess the non-tumoral liver. Our current analysis also has shortcomings, whereas the most important is the lack of an external cohort to verify our findings. This criticism is certainly justified, however we see the current work rather as a generation of hypothesis that the reading of imaging especially in the fragile context of liver function versus tumor control could be performed on a much more complex level than for example the BCLC staging. A further shortcoming is that some patients received prior treatment as Sorafenib^™^ (Bayer, Leverkusen, Germany) which might influence the outcome of PFS. The preceding treatments are summarized in Table 2 and reflect a standard population receiving radioembolization where this treatment is used rather in later therapy lines. However, prospective studies showed the feasibility and tolerability of anti-angiogenic treatment as Sorafenib followed by radioembolization [30]. This presented analysis is to our knowledge the first whole-liver radiomics approach, representing the fragile balance between liver function and tumor burden, which is the clinical reality in these patients. However, our results have to be verified in future prospective studies.

## MATERIALS AND METHODS

### Patient characteristics

All pretreatment ^18^F-FDG PET images of patients undergoing ^90^Y-TARE for uHCC between December 2010 and December 2015 were retrospectively analyzed. The American Association for the Study of Liver Diseases (AASLD) guidelines [31] were used to diagnose HCC and the BCLC staging system have been used to stage HCC [4]. Patients included in the study had unresectable HCC because of a locally advanced tumor, multifocal disease or PVI. Also, inclusion criteria consisted of patients with a liver-dominant or liver-only disease, an adequate hematologic, renal and hepatic function, a good (ECOG PS) <2 and a life expectancy >3 months and a Child-Pugh score ≤ B7. Exclusion criteria were an inadequate liver reserve (bilirubin >34 μmol/L, ascites), a Child-Pugh score > B7, a poor ECOG PS ≥ 2, distant metastases, a higher lung shunt fraction > 20%, an estimated lung absorbed dose of >30 Gray per session and 50 Gray in total and an uncorrectable extrahepatic flow on the pretreatment ^99m^technetium-macroaggregated albumin single-photon emission computed tomography (^99m^Tc-MAA SPECT/CT). All patients underwent imaging procedures and ^90^Y-TARE as standard care. The local Ethics Research Committee of the State of Vaud took into account the retrospective analysis of our database, approved the protocol (Number 2016–00640) and waived the need for patient informed consent for the study analysis.

### ^18^F-FDG PET

All patients underwent ^18^F-FDG PET/CT on a Discovery D690 TOF (GE HealthCare, Waukesha, WI) 50–70 minutes after a planned intravenous injection of 3.6 ± 0.4 MBq/kg of ^18^F-FDG. All patients fasted for at least 6 hours and blood glucose levels were less than 140 mg/dL before administration of ^18^F-FDG. A low-dose helical CT (120kV, 80–200mA) was first performed for anatomical correlation and attenuation correction. Then, whole-body emission images were acquired using 7 to 9 overlapping bed positions of 2 min each (starting from the top of skull and ending at the mid-thigh). Images were reconstructed using iterative protocols with body weight-normalized SUV computation.

### Radiomics features segmentation and extraction

All CT livers were semi-automatically segmented using The Medical Imaging Interaction Toolkit (MITK) workbench software [32] to generate a three-dimensional mask that was further incorporated and translated to the ^18^F-FDG PET images. Three-dimensional texture analysis was applied to the pretreatment ^18^F-FDG PET study using an open-source software Chang Gung Image Texture Analysis toolbox (CGITA) [33] implemented in Matlab 2015b (Mathworks Inc., Natick, MA). A total of 108 radiomics features from the three-dimensional segmented livers of ^18^F-FDG PET images were extracted according from following categories: SUV statistics, co-occurrence matrix, voxel alignment matrix, neighborhood intensity difference matrix, intensity size zone matrix, normalized co-occurrence matrix, voxel statistics, texture spectrum, texture feature coding co-occurrence matrix and neighborhood gray level dependence. Steps of the radiomics process are illustrated in Figure 1.

### ^90^Y-TARE procedure

The ^90^Y-TARE planning and procedure was made as previously described [34]. Briefly, before ^90^Y-TARE, all patients underwent a pretherapy SPECT/CT with intra-arterial administration of 120–180 MBq of ^99m^Tc-MAA. The required ^90^Y administered activity was calculated from partition model dosimetry as reported by Gnesin *et al*. [35]. ^90^Y-resin (SIR-Spheres^™^; SIRTex Medical, Sydney, Australia) or ^90^Y-glass (TheraSphere^™^; BTG Biocompatibles Ltd, Farnham, UK) microspheres were injected by a nuclear physician into a percutaneous catheter inserted into the femoral artery and directed to the selected hepatic artery. Patients with small-tumor volumes were preferentially addressed to ^90^Y-glass microspheres due to their higher specific ^90^Y activity and lower particle number aiming at avoiding lesion saturation and consecutive reflux to non-target volumes. A post-^90^Y-TARE SPECT/CT was performed to confirm the distribution of ^90^Y microspheres.

### Study endpoints

Study endpoints were PFS and OS. PFS was defined as time from the date of the ^90^Y-TARE until the date of the first occurrence of hepatic tumor progression based on imaging data with contrast-enhanced CT or MRI using Response Evaluation Criteria in Solid Tumors, distant recurrence, death or last known consultation (censored). OS was defined as time from the date of the ^90^Y-TARE until death from any cause or last known consultation (censored).

### Statistical analysis

The statistical analysis was performed with R software (The R Project for Statistical Computing, www.r-project.org, version 3.3.2) [32]. The packages in R used in the present study were “glmnet” [36], “Survival” [37], “ggplot2” [38], “caret” [39], “matcor” [40]. All continuous variables were checked for normality and described with conventional statistics. All continuous numeric data were centered and scaled from the mean and standard deviation. According to the Harrell guideline as the number of events should exceed the number of included covariates by at least 10 times in a multivariate analysis [41], an initial reduction of variables was necessary. To address this issue, highly correlated variables were removed (which were defined as a Spearman's correlation > 0.9). On the remaining variables, the least absolute shrinkage and selection operator (LASSO) Cox regression model [42, 43] which is suitable for the regression of high-dimensional data, was used to select the most useful prognostic features in the data set. The selected imaging features were then combined into a radiomics signature. For each patient, PFS and OS predictive scores based on ^18^F-FDG PET radiomics signature (pPET-RadScore) were computed through a linear combination of selected features weighted by their respective coefficients. Using X-tile software version 3.6.1 (Yale University School of Medicine, New Haven, Conn) [44], the optimal pPET-RadScore value to predict PFS and OS served as cutoff to separate high- and low-risk patients. Survival curves of the high-risk and low-risk groups were estimated using the Kaplan-Meier method and differences between subgroups were compared with the log-rank test. Using SPSS software (version 23, SPSS Inc., Chicago, IL, USA), the differences in demographic, clinical, pathological and treatment data between these two groups were compared by using χ^2^ test with Pearson's correction for discrete variables and t test or Mann-Whitney test for continuous variables. The influence of PFS and OS-pPET-RadScores, BCLC staging system and serum AFP level was investigated using a Cox proportional hazards model. Stratified analyses were performed to explore the potential association of the radiomics signature with the PFS and OS using subgroups within clinical-pathologic risk factors from the whole data set. For all statistical analyses, *P* values < 0.05 were considered statistically significant.

## Abbreviations

AASLD: American Association for the Study of Liver Diseases
AFP: Alpha-fetoprotein
BCLC: Barcelona-Clinic Liver Cancer
CGITA: Chang Gung Image Texture Analysis
CLIP: Cancer of the Liver Italian Program
CT: Computed tomography
FDG: Fluorodeoxyglucose
LASSO: Least absolute shrinkage and selection operator
MAA: Macroaggregated albumin
MITK: Medical Imaging Interaction Toolkit
MRI: Magnetic resonance imaging
OS: Overall survival
PET: Positron emission tomography
PFS: Progression-free survival overall survival
PVI: Portal vein invasion
Rad: Radiomics
SPECT: Single-photon emission computed tomography
SUV: Standardized uptake value
T/L: Tumor-to-liver
TARE: Transarterial radioembolization
TNM: Tumor-node-metastasis
uHCC: Unresectabble hepatocellular carcinoma.
